# Parent routines, child routines, and family demographics associated with obesity in parents and preschool-aged children

**DOI:** 10.3389/fpsyg.2014.00374

**Published:** 2014-04-29

**Authors:** Blake L. Jones, Barbara H. Fiese, The STRONG Kids Team

**Affiliations:** ^1^Department of Human Development and Family Studies, Purdue UniversityWest Lafayette, IN, USA; ^2^Department of Human and Community Development, Family Resiliency Center, University of Illinois at Urbana-ChampaignUrbana, IL, USA

**Keywords:** obesity, health, protective routines (PR), adequate sleep, limited screen time, mealtimes, bedroom televisions, preschool children

## Abstract

Many daily routines and behaviors are related to the prevalence of obesity. This study investigated the association between routines and behaviors that act as protective factors related to lower prevalence of obesity in parents (BMI ≥ 30 kg/m^2^) and overweight in preschool children (BMI ≥ 85th percentile). Socio-demographic characteristics were assessed in relation to protective routines (PRs), and prevalence of obesity/overweight data from 337 preschool children and their parents. The two PRs assessed with parents included adequate sleep (≥7 h/night) and family mealtime routine (scoring higher than the median score). The four PRs assessed in children included adequate sleep (≥10 h/night), family mealtime routine, limiting screen-viewing time (≤2 h/day of TV, video, DVD), and not having a bedroom TV. Overall, 27.9% of parents were obese and 22.8% of children were overweight, and 39.8% of the parents had both parent PRs, and only 11.6% of children had all four child PRs. Results demonstrated that several demographic factors were significantly related to the use of PRs for parents and children. The lack of PRs was related to increased risk for overweight in children, but not for obesity in parents. However, in the adjusted models the overall cumulative benefits of using PRs was not significant in children either. In the multivariate adjusted logistic regression models, the only significant individual PR for children was adequate sleep. In a path analysis model, parent sleep was related to child sleep, which was in turn related to decreased obesity. Overall, findings suggest that parent and child PRs, especially sleep routines, within a family can be associated and may play an important role in the health outcomes of both parents and children. Understanding the mechanisms that influence how and when parents and children use these PRs may be promising for developing targeted family-based obesity-prevention efforts.

## Introduction

The high prevalence of overweight and obesity among children and adults in the United States continues to be a major public health concern (Ogden et al., 2012, 2013).

Numerous factors have been identified as health-risk behaviors that are related to increases in obesity. Many of these factors are part of a family's daily routines such as mealtimes, sleep, and media use. Household routines are proposed to organize family life and have been found to be associated with health outcomes including obesity (Jacobs and Fiese, 2007; Fiese et al., 2012). In addition, shortened sleep duration (e.g., Chaput et al., 2006; Cappuccio et al., 2008; Taveras et al., 2008; Bell and Zimmerman, 2010), increased television viewing (e.g., Robinson, 2001; Dennison et al., 2002; Viner and Cole, 2005; Danner, 2008) and having a television in the bedroom (e.g., Barr-Anderson et al., 2008) have been connected to increased obesity in children and adults. These lifestyle behaviors and routines are rarely practiced in isolation and are typically associated with one another. For example, decreased sleep has been associated with increased screen-viewing (e.g., Owens et al., 1999; Thompson and Christakis, 2005), and having a bedroom TV (e.g., Mindell et al., 2009). In turn, having a bedroom TV has also been associated with other health-risk behaviors such as having fewer family meals (Barr-Anderson et al., 2008). This is a concern because frequent family mealtimes have been associated with positive health-related behaviors and decreases in overweight and obesity (e.g., Rollins et al., 2010; Hammons and Fiese, 2011; Wansink and van Kleef, 2013). In addition, for over a decade the (American Academy of Pediatrics, 2001, 2013) has recommended the removal of bedroom televisions and that parents limit television viewing (and only the use of quality programming) to no more than 2 h per day. These recommendations were based on evidence that these media routines were associated with negative health and psychosocial outcomes. Television in particular may have several negative pathways to influencing obesity, including replacing time that could be used for physical activity with a sedentary activity, being a distraction while eating, and being a primary source of advertisements for unhealthy food choices. Also, as previously mentioned, if television has been connected to decreased sleep then it may relate to obesity through interfering with the quality or quantity of sleep one receives (e.g., Owens et al., 1999; Thompson and Christakis, 2005; Chaput et al., 2006; Taveras et al., 2008).

Because daily routines are frequently practiced together it is important to consider the possible cumulative effects of such practices. One of the recent methods for examining the influence of combined effects from various risk factors are cumulative risk models (e.g., Wells et al., 2010; Evans et al., 2012; Suglia et al., 2012; Lee and Hicken, 2013). Cumulative risk models examine multiple risk exposure on obesity outcomes by converting potential health-risk behaviors and other associated demographic factors into dichotomous variables (e.g., the risk is present = 1; the risk is not present = 0) that are then summed. Although the cumulative risk approach is fairly straightforward, there have been gaps in understanding the implications and mechanisms related to examining outcomes in relation to additive risks (Evans et al., 2013). In addition, these models essentially weight each risk as being equal in its influence on obesity, which usually does not allow for an examination of how the variance within each risk influences obesity outcomes. Cumulative risk models also tend to be pessimistic at a conceptual level, focusing on how exposure to combined risks leads to negative outcomes.

In contrast to the focus on risks and negative factors that increase prevalence of obesity (such as cumulative risk models), some researchers have focused on examining positive routines that act as protective factors to decrease prevalence of obesity (e.g., Anderson and Whitaker, 2010; Haines et al., 2013). These researchers have focused on the potential of promoting positive health-related behaviors and daily routines as positive mechanisms of change relating to obesity and health. In a similar way to how cumulative risks can compound their influence on negative health outcomes, researchers have suggested that cumulative protective routines (PRs) can work together to help support positive health outcomes. For example, Anderson and Whitaker reported that children who got adequate sleep, ate dinner with their parents, and limited screen-viewing time, had a 40% lower prevalence of obesity than children who were exposed to none of those PRs. Although some studies are starting to examine the cumulative effects of protective behaviors (such as healthy routines) in adults or children, there remains a gap in understanding how and when these healthy routines are related to positive health outcomes and protect against the risk of obesity prevalence. In addition, little is known regarding how and when the PRs of parents are related to the PRs of their children. For the purpose of the current study, PRs are defined as daily family and personal routines that are generally associated with, and likely to be predictive of, an individual's overall health.

The objective of the current study was to identify the potential associations between parent PRs and child PRs, and to describe socio-demographic factors relating to differences in the use of PRs. Because socio-demographic factors have been associated with daily routines (Fiese et al., 2012) and obesity (Taveras et al., 2010; Ogden et al., 2013) it was also important to consider demographic variations in PRs. This study reports on potential socio-demographic differences related to two parent PRs (getting adequate sleep ≥7 h night and having frequent and family mealtime routines). We hypothesized that parent PRs would be positively related to child PRs, and that individual and cumulative PRs would be related to decreased prevalence of obesity in parents and overweight in preschool children. To test these hypotheses, we examined how sociodemographic factors, as well as two specific parent PRs, were related to exposure to the four child PRs that were targeted by Haines et al. (2013) (e.g., getting adequate sleep ≥10 h night; regular family dinnertimes; limiting screen-viewing time to 2 h per day or less; avoiding the placement of a TV in the child's bedroom). We then assessed how parent PRs were related to obesity prevalence in parents, and how child PRs were related to overweight prevalence in children. Finally, we compared the prevalence of cumulative parent PRs, and each individual parent PR, with child overweight and the use of cumulative and individual child PRs.

## Materials and methods

### Participants

This study included 337 preschool-age children, and a primary caregiver of each child. The study was approved by the Institutional Review Board at the University of Illinois at Urbana-Champaign. The sub-sample used in this study came from the STRONG Kids study (e.g., Harrison et al., 2011; Dev et al., 2013). The only inclusion criteria for children from the STRONG Kids study to be included in the current study were that children needed to have a measured height and weight by study staff, and they needed to have complete data for child and parent PRs, to be eligible for inclusion. Although the initial STRONG Kids study included data for 497 children, objective anthropometric measurements were only obtained for 407 of those children. Data about one or more PRs were missing from 70 of the 407 participants, therefore they were also excluded from the current study. The final subsample included 337 children and one primary caregiver from each family. When we compared the final subsample to those who were excluded based on inclusion criteria, parents with complete survey data and measured child BMIs, the 337 children included were more likely to have higher parent education, higher family incomes, and be Caucasian, and were less likely to have single parents.

### Procedure

#### Parent measures

Parents of preschool-age children were recruited from advertisements distributed to child care centers throughout east-central Illinois, USA. Interested parents contacted research staff to express interest in participation. After obtaining informed consent from each parent, parents completed an extensive survey about the health and daily PRs of themselves and their preschool-age children. The majority of parent surveys were completed online, but many parents chose to complete a paper version of the survey that was provided to them with prepaid postage materials and instructions for mailing them back to the researchers.

***Parent BMI***. Parent BMI scores were calculated by entering parents' self-reported weight (in pounds, converted to kilograms) and height (in inches, which were converted to meters) and data into the standard equation for BMI [((mass in kilograms) ÷ ((height in meters)^2^))]. Next, a dichotomous variable was created by separating BMI scores into obese (BMI ≥ 30 kg/m^2^) vs. non-obese parents (BMI < 30 kg/m^2^).

***Parent sleep***. Parents were asked, “on the average, how many hours did you sleep each night during the past 4 weeks?” These responses were then converted to a dichotomous variable that separated parents who slept for 7 h or more vs. those that obtained less than 7 h of sleep per night on average.

***Family mealtime routine***. *Family m*ealtime routine was measured using a five-item scale adapted from the Family Ritual Questionnaire dinnertime scale (Fiese and Kline, 1993) that asked parents to answer the following questions about their family mealtimes using a 1-to-5-point Likert scale (1 = “not true at all” and 5 = “very true”): (1) “In our family, mealtime is planned in advance;” (2) “Our family regularly eats the main meal together;” (3) In our family, everyone is expected to be home for the main meal;” (4) “In our family at mealtime, everyone has a specific role or job to do;” and (5) “In our family, mealtime is flexible; people eat whenever they want.” Item 5 was reverse coded. The cronbach's alpha for the 5-item scale was 0.753, and higher scores on the scale represented a higher sense of positive mealtime climate associated with the family mealtime routine. The median score for the family mealtime routine scale was 3.83, and was then used to create a dichotomous variable representing high and low mealtime routine scores. Scores at the median or higher were labeled as higher family mealtime routine, and those below the median were labeled as lower family mealtime routine.

#### Child measures

After each parent completed the survey, research staff coordinated times with parents so that the trained staff members could collect anthropometric measurements of child height and weight from each child. All other information for these preschool children came from parent-report survey data.

***Child overweight***. Child overweight status was measured using standard procedures recommended by the Centers for Disease Control (CDC, 2012). The CDC procedures for measuring height and weight specify that trained researchers measure each height and weight twice, to the nearest 0.1 cm and 0.1 kg using a stadiometer and a digital scale. The measurements are then averaged, and the information for each individual is entered into the CDC's BMI Tool for Schools program, which calculates BMI scores and percentiles based on child BMI-for-age growth charts. Child BMI percentiles were then converted to a dichotomous variable with children under the 85th percentile grouped together as “healthy” weight status. Children with BMI scores at the 85th percentile or above were grouped together to represent those who were overweight (or “at-risk-for-overweight”).

***Child sleep***. Parents responded to the following question about children's sleep, “during the past week, how many hours of sleep did your child get each night (on average)?” Child sleep hours were then broken into a dichotomous variable representing children who obtained 10 or more hours of sleep vs. those that obtained less than 10 h of sleep on an average night.

***Family mealtime routine***. Each parent completed one overall mealtime routine score for the family. Therefore, the child in each family had the same mealtime routine status (high or low) as the parent (as previously described). Because both the parent and the child had the same score on that routine, the analyses were constructed so that the parent and child mealtime routine status were never used in the same analysis in comparison (see Table 5 for example).

***Child limited screen viewing time***. Parents reported the number of minutes that their children viewed television, videos, and DVDs each day on average. The times for all screen-viewing time were then combined and converted into the number of hours per day that children spent in screen-viewing time. Finally, a dichotomous variable was created to distinguish children who limited screen-viewing time to 2 h per day or less vs. those that viewed more than 2 h per day.

***Child not having a bedroom TV***. Parents reported whether the children had a television in the room they slept in, resulting in a dichotomous “yes” (have a bedroom TV) or “no” (do not have a bedroom TV) variable.

## Results

The mean age of children was 3.22 years (*SD* = 0.64 years, range 2–4 years), and mean age of parents was 32.37 years (*SD* = 6.67 years, range 20–64 years). The study included 178 preschool boys (53.0%) and 158 preschool girls (47%; with one child's gender not reported). The majority of the parents that participated were mothers (*n* = 302), but some fathers participated as well (*n* = 34; with 1 parent gender missing). Parents were generally highly educated and the average annual family income was $59,736 (*SD* = $36, 541). The median income was $55,000, which is slightly higher than the U.S. average median income of $51,000 (U.S. Census Bureau, 2014). Over half of the participants were non-Hispanic white (60.4%), and 25.6% lived in a single-parent household (Table 1). The prevalence of obesity among parents was 27.9%, and the prevalence of overweight among preschool children was 22.8%.

**Table 1:** **Sociodemographic characteristics among STRONG Kids participants**.

**Sociodemographic characteristic**	**Unweighted, *n***	**Prevalence, %**
**PARENT EDUCATION^a^**		
High school or less	34	10.1
Some college or technical school	104	30.9
Bachelor's degree	104	30.9
Graduate school	95	28.2
**PARENT GENDER^b^**		
Men	34	10.1
Women	302	89.9
**SINGLE-PARENT HOUSEHOLD^c^**		
No	247	74.4
Yes	85	25.6
**RACIAL/ETHNIC GROUP^d^**		
Non-hispanic white	203	60.4
Non-hispanic black	78	23.2
Hispanic	16	4.8
Other	39	11.6
**MATERNAL OBESITY^e^**		
Maternal BMI < 30 kg/m^2^	233	72.1
Maternal BMI ≥ 30 kg/m^2^	90	27.9
**CHILD OBESITY**		
Child BMI < 85th percentile	260	77.2
Child BMI ≥ 85th percentile	77	22.8
**FAMILY INCOME^f^**		
$24,999 or less	75	24.0
$25,000–39,999	44	14.1
$40,000–69,999	62	19.9
$70,000–99,999	62	19.9
$100,000 or more	69	22.1

*Sample sizes are unweighted, with a total N = 337 participants*.

a *Parent education has missing data from 1 participant*.

b *Parent gender has missing data from 1 participant*.

c *Single-parent household status has missing data from 5 participants*.

d *Race has missing data from 1 participant*.

e *Maternal obesity has missing data from 9 participants*.

f *Family income has missing data from 25 participants*.

Additional analyses were conducted to compare bivariate correlations between parent and child weight status and health outcomes in relation to each of the parent and child PRs and some of the socio-demographic factors (see Table 2). Parent obesity and child overweight were significantly related. Although the correlations between individual and overall routines were low to moderate, several correlations were statistically significant. Parent sleep was not significantly related to family mealtime routines. All of the child PRs were correlated to one another, with the strongest being child sleep and limiting screen viewing. Parent sleep had a small, significant correlation with children not having a bedroom TV. Although the dichotomous variables of parent sleep and child sleep were not correlated, the actual hours of parent sleep (*M* = 7.11 h, *SD* = 1.07) and child sleep (*M* = 9.38, *SD* = 1.15) were correlated (*r* = 0.18, *p* < 0.01).

**Table 2:** **Bivariate correlations between obesity/overweight prevalence, health, protective routines, and family demographics**.

**Variable**	**Mean**	***SD***	**1**	**2**	**3**	**4**	**5**	**6**	**7**	**8**	**9**	**10**	**11**
1. Parent prevalence of obese, %^a^	27.86	44.90	—										
2. Child prevalence of overweight, %^b^	22.85	42.05	0.19^**^	—									
3. Parent sleep ≥ 7 h/d,%	73.29	44.31	−0.02	0.01	—								
4. Family mealtime routine high, %	54.01	49.91	−0.07	0.01	0.01	—							
5. Child sleep ≥ 10 h/d, %	51.93	50.04	−0.11^+^	−0.23^**^	0.08	0.19^**^	—						
6. Child limits screen time ≤ 2 h/d, %	46.59	49.96	−0.17^**^	−0.14^**^	0.05	0.18^**^	0.30^**^	—					
7. No TV in child bedroom, %^c^	34.72	47.69	−0.16^**^	−0.09	0.13^*^	0.10^+^	0.14^**^	0.26^**^	—				
8. Total # protective parent routines^d^	1.27	0.67	−0.06	−0.01	0.67^**^	0.75^**^	0.19^**^	0.17^**^	0.16^**^	—			
9. Total # protective child routines^e^	1.87	1.24	−0.20^**^	−0.18^**^	0.11^+^	0.59^**^	0.65^**^	0.70^**^	0.58^**^	0.51^**^	—		
10. Family income	$59,736	$36,541	−0.26^**^	−0.12^*^	0.15^**^	0.17^**^	0.25^**^	0.23^**^	0.25^**^	0.22^**^	0.36^**^	—	
11. Parent education^f^	2.77	0.97	−0.24^**^	−0.20^**^	0.09	0.20^**^	0.25^**^	0.24^**^	0.19^**^	0.21^**^	0.35^**^	0.59^**^	—

*N = 337*.

** p < 0.01,

* p < 0.05,

+ *p < 0.10*.

a *Parent BMI ≥ 30 kg/m^2^*.

b *Child BMI ≥ 85th percentile*.

c *The mean for child bedroom television prevalence is the percentage of children who do not have a bedroom television*.

d *Two parent protective routines include (1. sleep ≥ 7 h/night; 2. high family mealtime routine score)*.

e *Four child protective routines include (1) sleep ≥ 10 h/night; (2) high family mealtime routine score; (3) limit screen viewing ≤ 2 h per day of TV, DVD, and video, and (4) child does not have a bedroom television*.

f *Parent education is taken from a categorical variable (1) high school diploma or less, (2) at least some college or technical school training, (3) bachelor's degree, (4) grad school or higher*.

ANOVA tests were conducted to examine potential differences in demographic characteristics that related to whether or not PRs were present in parents and children. These tests were used to assess differences between parent and child PRs in relation to socio-demographic characteristics such as parent education, single-parent households, racial/ethnic groups, and family income (see Tables 3, 4). All four of the demographic characteristics were associated with differences in the prevalence of parent obesity and child overweight. In general, there were also significant differences across demographic variables on each of the parent and child PRs so that increases in PRs were related to higher parent education, parents being married, higher family income, and higher overall parent health, and varied by race. For ANOVA tests with three or more groups, *post-hoc* analyses were conducted (using Tukey HSD). Overall, these analyses revealed that the most significant differences in PRs were from the highest and lowest of the demographic categories (parent education and family income). The results generally supported linear relationships between PRs with increased incomes and education.

**Table 3:** **Prevalence of parent obesity and protective routines according to sociodemographic characteristics**.

**Characteristic**	**Prevalence of parent obesity, %^a^**	**Parent sleep ≥7 h/night, %**	**Mealtime routine high, %**	**Parent has both protective routines, %**
**PARENT EDUCATION LEVEL**				
High school or less	37.5	70.6	35.3	26.5
Some college	43.0	68.3	46.2	31.7
Bachelor's degree	22.0	74.0	56.7	42.3
Graduate school or more	14.3	79.0	66.3	50.5
*F*^b^	10.860^**^	1.280	11.755^**^	7.692^**^
**SINGLE-PARENT HOUSEHOLD**				
No	24.2	75.7	59.1	44.1
Yes	39.0	67.1	38.8	28.2
*F*	6.779^**^	2.432	10.750^**^	6.748^**^
**RACIAL/ETHNIC GROUP**				
Non-hispanic white	26.4	77.3	60.1	45.8
Non-hispanic black	40.5	62.8	29.5	20.5
Hispanic	31.3	68.9	68.8	37.5
Other	8.6	74.4	66.7	48.7
*F*	4.343^**^	2.097^+^	9.219^**^	14.248^**^
**ANNUAL FAMILY INCOME**				
$24,999 or less	44.6	62.7	44.0	26.7
$25,000–39,999	40.0	72.7	45.5	36.4
$40,000–69,999	25.4	71.0	50.0	33.9
$70,000–99,999	19.7	75.8	62.9	46.8
$100,000 or more	13.9	82.6	63.8	53.6
*F*	21.842^**^	6.939^**^	8.798^**^	7.201^**^

a *Obesity defined as parents with BMI ≥ 30 kg/m^2^*.

b *Unweighted between group ANOVA tests*.

** p < 0.01,

^*^p < 0.05,

+ *p < 0.10*.

**Table 4:** **Prevalence of child obesity and protective routines according to sociodemographic characteristics**.

**Characteristic**	**Child at-risk-for-overweight, %^a^**	**Child sleep ≥ 10 h/night, %**	**Mealtime routine high, %**	**Child limiting screen-viewing %**	**Child does not have a bedroom television, %**	**Child has all 4 protective routines, %**
**PARENT EDUCATION LEVEL**						
High school or less	38.2	29.4	35.3	35.3	26.5	0.0
Some college	30.8	41.4	46.2	31.7	22.1	4.8
Bachelor's degree	18.3	56.7	56.7	49.0	39.4	13.5
Graduate school or more	13.7	66.3	66.3	64.2	46.3	21.1
*F*^b^	11.465^**^	17.693^**^	11.755^**^	12.189^**^	7.114^**^	13.883^**^
**SINGLE-PARENT HOUSEHOLD**						
No	19.0	60.7	59.1	51.4	39.7	13.4
Yes	31.2	25.9	38.8	32.9	21.2	5.9
*F*	5.994^*^	33.691^**^	10.750^**^	8.852^**^	9.743^**^	3.505^+^
**RACIAL/ETHNIC GROUP**						
Non-hispanic white	23.7	59.6	60.1	52.7	37.4	14.3
Non-hispanic black	29.5	28.2	29.5	29.5	20.5	2.6
Hispanic	12.5	56.3	68.8	25.0	43.8	12.5
Other	7.7	59.0	66.7	59.0	46.2	15.4
*F*	2.742^*^	8.237^**^	9.219^**^	6.123^**^	3.541^*^	2.765^*^
**ANNUAL FAMILY INCOME**						
$24,999 or less	32.0	32.0	40.0	38.7	22.7	4.0
$25,000–39,999	20.5	45.6	45.5	31.8	22.7	4.6
$40,000–69,999	22.6	58.1	50.0	41.9	27.4	9.7
$70,000–99,999	22.6	62.9	62.9	45.2	43.6	12.9
$100,000 or more	14.5	65.2	63.8	71.0	52.2	23.2
*F*	4.771^*^	19.954^**^	8.798^**^	17.252^**^	20.086^**^	15.333^**^
**PARENT OBESITY**						
Parent BMI < 30 kg/m^2^	17.7	55.5	53.6	51.6	39.5	13.7
Parent BMI ≥ 30 kg/m^2^	34.6	39.4	48.5	22.9	21.4	5.4
*F*^b^	11.633^**^	3.634^+^	1.404	9.658^**^	7.960^**^	8.278^**^

a *Obesity defined as parents with BMI ≥ 30 kg/m^2^*.

b *Unweighted ANOVA tests*.

** p < 0.01,

* p < 0.05,

+ *p < 0.10*.

Logistic regression models were used to estimate the odds of parent obesity associated with the exposure to each parent individual PR and the total number of PRs used by parents. These analyses yielded unadjusted odds ratios (ORs), as well as multivariate adjusted ORs that controlled for parent education, race/ethnicity, parent gender, parent age, and single-parent households (see Table 5). Overall, 39.8% of the parents both parent PRs, and 12.5% of parents did not have either of the parent PRs. Individual and cumulative parent PRs were not significantly related to a lower prevalence of obesity in adults. For children, 32.3% had three or four of the PRs. These numbers were added together because only about 11.6% of the children had all four PRs and there was not a significant benefit from having all four PRs vs. any of the three PRs. In fact, having three PRs was associated with lower BMI outcomes in the unadjusted and adjusted logistic regression models. Our analyses indicated that exposure to the two parent PRs were not significantly related to a lower prevalence of parental obesity in the unadjusted model, or when controlling for demographic factors. In children the lack of PRs was related to increased risk for overweight in the unadjusted models, but the increased risks (in ORs) were not significant when controlling for demographic factors. The only individual PR that remained significant in the multivariate adjusted model was children getting at least 10 h of sleep per night.

**Table 5:** **Associations between protective routines and obesity for parents and children**.

**No. of protective routines**	**Has routine or not, % (95% CI)**	**Obesity prevalence, % (95% CI)^a^**	***F*^b^**	**OR**	**(95% CI)**
				**Unadjusted**	**Multivariate adjusted^c^**
**PARENT # OF ROUTINES**					
Both routines	39.8 (34.5–45.0)	24.6 (17.1–32.1)	0.776	1.00 (reference)	1.00 (reference)
Only 1 routine	47.8 (42.4–53.1)	29.6 (22.3–37.0)		1.29 (0.76–2.19)	1.14 (0.65–2.00)
No routines	12.5 (08.9–16.0)	31.7 (16.8–46.6)		1.42 (0.66–3.07)	1.40 (0.63–3.09)
**SPECIFIC PROTECTIVE PARENT ROUTINES**					
Parent sleep ≥ 7 h/d	73.3 (68.6–78.0)	27.4 (19.3–38.9)	0.084	1.00 (reference)	1.00 (reference)
Lacks routine	26.7 (22.0–31.5)	29.1 (21.7–33.4)		1.08 (0.63–1.87)	1.11 (0.59–2.08)
Mealtime routine	54.0 (48.7–59.4)	25.1 (18.7–31.6)	1.404	1.00 (reference)	1.00 (reference)
Lacks routine	46.0 (40.7–51.3)	31.1 (23.5–38.6)		1.34 (0.83–2.19)	1.15 (0.64–2.07)
**CHILD ROUTINES**					
Any 3 or all 4^d^	32.3 (27.3–37.4)	13.8 (7.2–20.3)	10.550^**^	1.00 (reference)	1.00 (reference)
Any 2	27.0 (22.2–31.8)	19.8 (11.4–28.1)		1.55 (0.73–3.27)	1.18 (0.45–3.09)
Only 1	24.6 (20.0–29.3)	31.3 (21.1–41.5)		2.86 (1.40–5.85)^**^	1.47 (0.47–4.54)
None	16.0 (12.1–20.0)	33.3 (20.4–46.3)		3.13 (1.43–6.87)^**^	1.97 (0.81–4.79)
**SPECIFIC PROTECTIVE CHILD ROUTINES**					
Child sleep ≥ 10 h/d	51.9 (46.6–57.3)	13.7 (8.6–18.9)	18.052^**^	1.00 (reference)	1.00 (reference)
Lacks routine	48.1 (42.7–53.4)	32.7 (25.4–40.0)		3.06 (1.78–5.26)^**^	2.87 (1.50–5.49)^**^
Mealtime routine	54.0 (48.7–59.4)	23.1 (16.9–29.3)	0.012	1.00 (reference)	1.00 (reference)
Lacks routine	46.0 (40.7–51.3)	22.6 (15.9–29.2)		1.03 (0.62–1.71)	1.28 (0.69–2.39)
Limits screen time	46.6 (41.2–51.9)	16.6 (10.7–22.4)	6.685^**^	1.00 (reference)	1.00 (reference)
Lacks routine	53.4 (48.1–58.8)	28.3 (21.7–35.0)		1.99 (1.17–3.39)^*^	1.30 (0.70–2.42)
No bedroom TV	34.7 (29.6–39.8)	18.0 (10.9–25.0)	2.444	1.00 (reference)	1.00 (reference)
Lacks routine	65.3 (60.2–70.4)	25.5 (18.3–27.4)		1.56 (0.89–2.74)	1.05 (0.53–2.07)

a *Obesity prevalence for adults was BMI ≥ 30 kg/m^2^, for children it was BMI ≥ 85th percentile*.

b *Unweighted ANOVA tests*.

c *Parent and child routines were adjusted for parent gender, parent age, racial/ethnic group, family income, parent education, and single-parent household (child were also adjusted for parent BMI)*.

d *Child routines were combined for three and four routines because only 11.6% of children had all 4 routines and it did not make a large enough percentage to use as a reference group category*.

** p < 0.01,

* p < 0.05,

*^+^p < 0.10*.

**Figure 1:**
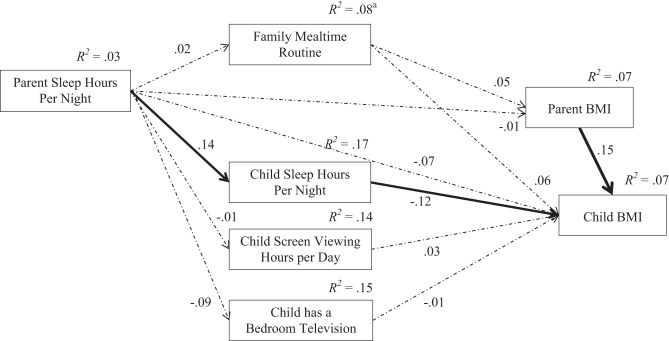
**Protective parent and child routines and obesity outcomes**. The overall model (*N* = 337) fit the data well, χ^2^_(23)_ = 16.200, *p* = 0.847, CFI = 1.000, TLI = 1.000, RMSEA = 0.000. ^a^The model was adjusted for parent age, parent gender, child age, child gender, race, family income, parent education, and single parent households; and the *R*^2^-values for PRs at the left and center of the model reflect the influences from control variables that are not included in the model shown for parsimony. The solid lines are significant at *p* < 0.05, and the dashed lines are not significant in the adjusted model.

Finally, a path analysis model was constructed using structural equation modeling (SEM) with Analysis of Moment Structures (AMOS) software version 21.0 (Arbuckle, 2013). The path model (see Figure 1) was used to analyze the individual and covariate relationships between the PRs with both parent obesity and child overweight. The model was designed to assess the connections between parent routines and child routines with the obesity outcomes for parents and children. The path model used the continuous data for the family mealtime routine, parent and child sleep hours, child screen viewing hours, and BMI scores. The overall analysis showed excellent fit to the data for model, [*n* = 337, χ^2^_(23)_] = 16.200, *p* = 0.847, CFI = 1.000, TLI = 1.000, RMSEA = 0.000. After controlling for the same demographic variables in the multivariate adjusted logistic regression models, the path model showed that parent sleep was related to child sleep, and that both child sleep and parent BMI were significantly related to child overweight.

## Discussion

Our analyses indicated that the cumulative effects of PRs were generally not associated with increases in prevalence for obesity in adults or overweight for children when controlling for demographic factors. The only individual PR that significantly decreased the risk for overweight in children was getting adequate sleep. Children who did not get adequate sleep had a greater risk for overweight than children who used at least three PRs regularly (*OR* = 2.87), even after controlling for parental BMI and socio-demographic characteristics. This finding suggests that sleep may be one of the most important routines to target in child obesity interventions. A recent study found that of the 22 risk factors that had been identified as significant predictors of child obesity, child sleep was the most significant predictor of child obesity (Dev et al., 2013). Although it is plausible to consider sleep as a sedentary behavior that would seem to be associated with higher prevalence of obesity, research has clearly shown that sleep in adults and children is strongly related to obesity and health outcomes (e.g., Chaput et al., 2006; Cappuccio et al., 2008; Taveras et al., 2008; Bell and Zimmerman, 2010). Because sleep habits and routines may persist from childhood into adulthood, it is important to focus on sleep routines in early childhood as a potentially effective obesity intervention strategy. As Haines et al. (2013) demonstrated, interventions targeting child sleep duration can effectively influence positive and protective behavior change. Future studies should continue to focus on understanding how both child and adult sleep routines relate to obesity and health outcomes, and which mechanisms affect how and when sleep routines provide the most beneficial influence on obesity and health outcomes.

Because targeted interventions are not likely to change influential sociodemographic characteristics such as parent education and family income, it is helpful to go beyond controlling for these variables and examine how variation of PRs within these variables. Therefore, it was important to identify when and how sociodemographic characteristics related to the presence of PRs. In general, parent education, family income, and single-parent households were related to the amount of parent PRs and child PRs that each family practiced in this sample. This information, as well as how PR prevalence differed by race, gender, or age, are helpful in informing how to target interventions for each specific group that practitioners and researchers are working with in interventions. Additionally, although efforts to increase the prevalence of children who get adequate sleep would be important for all children, this study suggests that it may be especially helpful for non-Hispanic Black children, who had the lowest prevalence of getting adequate sleep (only 28% compared to more than 56% for all other groups).

Some of the results of the current study were surprising. For example, in the analyses and path analysis model it was interesting that family mealtimes were not related to obesity for parents or children when this has been well-established in other studies (e.g., Hammons and Fiese, 2011; Wansink and van Kleef, 2013). Also, although parent sleep was significantly related to child sleep, it was not significantly related to parent BMI or child BMI in this population. This was surprising because most of the previous literature has shown strong connections between sleep and BMI in adults and children (e.g., Chaput et al., 2006; Cappuccio et al., 2008), and sleep was only related to obesity for the children in this study. These may have been related to the specific sample that these data came from, for example because the parents had increased education in general they may have more established sleep and mealtime routines than some other populations. Therefore, those routines may not have affected the parents as much in their personal health than some other studies have found.

The connection between parent and child sleep was important (especially as shown in the path analysis model), particularly because it demonstrates that it may be beneficial to give consideration toward parent routines when trying to develop targeted interventions to address or modify child routines. Another example of this possible connection was the relation between children's bedroom televisions and screen time with parents' sleep hours and BMI scores. Although this dataset did not include parents' screen time or bedroom televisions, it may be that those children who have more exposure to media in both ways are in homes where parents view more screen time and have bedroom TVs of their own. For these reasons it is very important for future researchers to include these variables for parents in their studies and to examine the connection between mealtimes, media, sleep, and obesity between parents and children.

This study offers several strengths and additions to the current literature about PRs. The comparison of both parent PRs and child PRs is unique, and is an important consideration for studies relating to routines. This study also examines the presence of PRs in connection to variations in sociodemographic characteristics, as well as how parent PRs relate to child PRs. These analyses allow for a more complete understanding of how PRs from one family member are related to other family members' routines. It is important to emphasize that routines in a household typically affect multiple members, not just children (Fiese and Jones, 2012). When designing interventions that will seek to change the behaviors of preschool children, it is critical to have parents be fully engaged in the process because they are the most influential regulators of these behaviors in such young children. Although they may not ultimately be able to control the exact amount or quality of sleep their child gets, they can help establish healthy sleep routines that encourage and promote the conditions for ideal sleep. Even though child behaviors influence how difficult it is to maintain mealtime routines and can affect the overall climate of the mealtime routine, it is primarily parents who ultimately decide the frequency of mealtimes and the quality of the food and food environment in the home. The same can be said for media use and the presence of televisions in children's bedrooms. For these reasons, it may be most beneficial for intervention studies to design obesity interventions as a family affair. For example, the parent and child can have goals for how to promote positive behavior change together by focusing on the sleep habits of both parent and child, or to focus on media use together. Though it may be hard to convince a child to allow a parent to remove a bedroom television once it is there, it could be seen as a show of solidarity and effort if the parent was willing to remove a television from their own bedroom, or limit their own screen time in efforts to spend that time with the child engaged in more constructive or healthful activities. This may also be a good place to start for many families because bedroom televisions are perhaps one of the most easily modifiable risk behaviors for obesity. In just a few minutes a television can be removed, although as other researchers have noted this change often involves resistance from even young children (e.g., Jordan et al., 2006).

This study included several limitations as well. Although the child BMI scores were derived from measured heights and weights, the remainder of the data were based on parent reports rather observations. It is important for future research that considers parent and child routines to have measured BMI scores for both children and parents. Also, response bias is certainly a concern. The population for this study reported slightly higher education, family income, and married status than the general population in the U.S., but was more diverse in race and ethnicity than the general U.S. population. The overall rates of overweight and obesity are also slightly lower than the U.S. general population, therefore using this somewhat homogenous sample it is important to note that generalizability to the entire U.S. population is limited. Also, the data did not include specific details about the context or variation within some of the routines. For example, we do not know about the sleep quality of the parents or children, including factors such as night-time waking or time parents and children went to bed or awoke from bed. These factors are important because recent research has shown that late bedtimes are related to increased calorie intake and screen time (both risks for obesity), even after controlling for sleep duration (Adamo et al., 2013). The dataset did not include information about parents' use of screen-viewing time or the presence of televisions in parents' bedrooms. Including parental media habits could be important for understanding not only their influence on parent obesity, but also their impact on child routines and ultimately child obesity. Also, the data about mealtimes were self-report, and did not reflect the quality of interactions during the meals or other dynamics such as food preparation or quality, or whether there was a television on in the background. Future research would benefit from identifying the context of the mealtimes to be able to assess how the quality and length of the mealtime and the mealtime interactions are related to other routines and obesity outcomes. Finally, as noted in the method section the participants in this study who had complete data were more likely to be from homes with higher parental education and family income. These demographic differences in this study limit generalizability further, but also highlight the need for future researchers to focus additional efforts on obtaining completed information from participants. This is especially important when collecting data from populations that may be at higher risk for obesity and other health outcomes.

After controlling for socio-demographic factors, obesity prevalence was only significantly related to children's sleep duration. Although some specific PRs are related to larger decreases in obesity prevalence directly, socio-demographic differences accounted for much of the significant variance. For this reason it is important for future interventions to consider the socio-demographic context when developing and implementing targeted intervention strategies to help prevent or decrease obesity. While the cumulative effects of PRs may have some additive effects that benefit health, they were not clearly seen in this study. Rather, this study demonstrated the individual connections between some parent and child PRs that may provide an important gateway for future research to identify effective targeted strategies for intervention. Overall, this study supports previous research that suggests that PRs are an important and effective component to consider when studying obesity. By identifying how and when socio-demographic variables and other factors influence the relationship between routines and obesity outcomes, researchers will be better informed in their efforts to design targeted obesity prevention and intervention programs.

### Conflict of interest statement

The authors declare that the research was conducted in the absence of any commercial or financial relationships that could be construed as a potential conflict of interest.
